# Priorities for global research into children’s palliative care: results of an International Delphi Study

**DOI:** 10.1186/s12904-015-0031-1

**Published:** 2015-08-04

**Authors:** Julia Downing, Caprice Knapp, Mary Ann Muckaden, Susan Fowler-Kerry, Joan Marston, on behalf of the ICPCN Scientific Committee

**Affiliations:** International Children’s Palliative Care Network, Assagay, South Africa; Makerere University, PO Box 7072, Kampala, Uganda; Pennsylvania State University, University Park, State College, PA 16801 USA; Tata Memorial Hospital, Mumbai, Maharashtra India; University of Saskatchewan, Saskatoon, SK Canada

**Keywords:** International, Delphi study, Children’s palliative care, Research

## Abstract

**Background:**

There is an urgent need to develop an evidence base for children’s palliative care (CPC) globally, and in particular in resource-limited settings. Whilst the volume of CPC research has increased in the last decade, it has not been focused on countries where the burden of disease is highest. For example, a review of CPC literature in sub Saharan Africa (SSA) found only five peer-reviewed papers on CPC. This lack of evidence is not confined to SSA, but can be seen globally in specific areas, such as an insufficient research and evidence base on the treatment of pain and other symptoms in children. This need for an evidence base for CPC has been recognised for some time, however without understanding the priorities for research in CPC organisations, many struggle with how to allocate scarce resources to research.

**Method:**

The International Children’s Palliative Care Network (ICPCN) undertook a Delphi study between October 2012 and February 2013 in order to identify the global research priorities for CPC. Members of the ICPCN Scientific Committee formed a project working group and were asked to suggest areas of research that they considered to be important. The list of 70 areas for research was put through two rounds of the Delphi process via a web-based questionnaire. ICPCN members and affiliated stakeholders (n = 153 from round 1 and n = 95 from round 2) completed the survey. Participants from SSA were the second largest group of respondents (28.1 % round 1, 24.2 % round 2) followed by Europe.

**Results:**

A list of 26 research areas reached consensus. The top five priorities were: Children’s understanding of death and dying; Managing pain in children where there is no morphine; Funding; Training; and Assessment of the WHO two-step analgesic ladder for pain management in children.

**Conclusions:**

Information from this study is important for policy makers, educators, advocates, funding agencies, and governments. Priorities for research pertinent to CPC throughout the world have been identified. This provides a much needed starting place for the allocation of funds and building research infrastructure. Researchers working in CPC are in a unique position to collaborate and produce the evidence that is needed.

## Background

Palliative care should be available to all children who need it, regardless of where they live, their culture, nationality, or stage of illness. Yet there are at least an estimated seven and a half million children who need palliative care [1] and in many parts of the world, children’s palliative care (CPC) is unavailable or limited to only a few services. Even when palliative care is available it is often not adapted to the needs of children [2]. Several international organisations, such as the International Children’s Palliative Care Network (ICPCN) and the Union Internationale Contre le Cancer (UICC) believe that every child with a life-limiting illness has the right to a high standard of total care, wherever they live in the world, and that the provision of palliative care for children is a global health issue [3]. This was endorsed by the recent World Health Assembly resolution which recognised the importance of CPC and member states committed to developing such services [4].

It has been well documented that there are many challenges to the provision of palliative care for children including a lack of education for families and providers; limited resources; lack of evidence; limited access to medications - including opioids for pain treatment- and the lack of appropriate policies to ensure availability and access within the health care system [2, 5, 6].

As the demand for CPC increases globally, there is a need to develop an evidence base in order to strengthen services [7]. This is particularly important in resource-limited settings where the burden of disease is high when compared with more developed countries and very little research has been conducted in resource-poor countries. Harding and colleagues emphasised the need for an increase in palliative care research through collaboration in low resource settings in order to address the needs of patients [8].

Moreover, there is a desperate need to establish methodologically robust evidence on how best to deliver palliative care in resource-limited settings [9]. This was highlighted in a review of palliative care in sub-Saharan Africa (SSA) where only five peer-reviewed papers were found [10] and has been echoed in other resource limited settings [11]. This lack of evidence has been attributed to a lack of locally relevant and validated tools to measure outcomes in CPC, lack of adequate sample sizes, and hesitancy to conduct research with dying children [12]. Overall, this lack of evidence on CPC is a global issue. Even in an area where some information is known, such as evidence on the treatment of pain in children, there still remains many unanswered questions and children still suffer from unnecessary pain during their lifespan. Through collaboration, the global CPC community could help develop informational resources and guidelines on best practice and produce the much needed evidence [13]. This need for collaboration was recognised in the ICPCN Declaration of Cape Town, where practitioners working in CPC agreed to collaborate together to improve the quality of palliative care for children [14]. Moreover, there is a need to develop a multi-stakeholder approach to research that enables those caring for children on a day-to-day basis, including families, to be part of the development of evidence. Most importantly, research into CPC needs should be focused on the child regardless of aetiology and consider the vulnerable nature of that child and his family [15].

Three studies have looked at research priorities in CPC at the national level, in Scotland [16, 17], Canada [18] and the United States of America [19]. All three of these studies consulted professionals working in the field, with two also addressing the views of parents and family members [16, 17, 19]. Whilst done in different settings, and acknowledging the differences between the health care systems and the development of CPC in these countries, there were similarities in the findings. Malcolm et al. [16, 17] in their study in Scotland identified the three key priority areas for research as being the hospice and respite care needs of young people, pain and symptom management, and bereavement and end-of-life care. In Canada, Steele et al. [18] also highlighted a research priority into what matters most for patients and parents receiving palliative care, alongside priorities in pain and symptom management, bereavement and end-of-life care. More recently, Baker et al. [19] in the US grouped their identified research priorities into the areas of decision-making (with the top priority being decision making at the end-of-life), care co-ordination, symptom management, quality improvement, and education.

Thus, whilst these studies [16–19] have identified research priorities for CPC at the national level, this has not been done internationally. To address this gap in information ICPCN undertook a Delphi study in order to identify the global research priorities for CPC. As an international collaboration of experts, the ICPCN Scientific Committee was in a unique position to take the lead on this study, and then to use the findings to develop and recommend an international research agenda for CPC to ICPCN and other organisations. Results from the study can also be used to inform funders and provide an evidence base to justify research proposals.

## Methods

The Delphi method was used to identify and prioritise areas for research into CPC [20] as this method has been previously successful in identifying national priorities in CPC [16–19] as well as in international health research [21–25]. To start the Delphi process a working group of 20 individuals consisting of the ICPCN Scientific Committee and expert advisers, was formed. Members were selected based on their expertise as specialists in CPC and their expertise in research. Ethical approval for the study was gained from the Uganda National Council for Science and Technology (Ref HS 1035).

The four steps of our Delphi study are described below.

- Step 1: Baseline list of priority areas for research into children’s palliative care

**Fig. 1:**
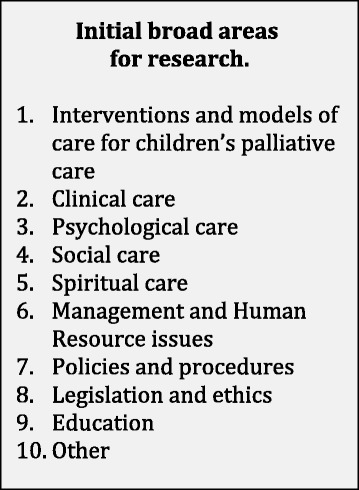
Initial broad areas for research

Members of the working group were asked to suggest areas of research that they considered to be important in CPC. An initial list of broad themes for research was provided based on the toolkit for CPC [26] (Fig. 1). The toolkit, developed in order to share knowledge, policies and practices that have proven effective in developing CPC services in the African setting, was used to form the initial outline as it covered the key areas for CPC practice and development, including issues encountered in resource-limited settings. Members of the working group were asked to suggest priorities under each of the broad themes along with additional areas that they considered to be priorities for research in the field. Participants completed written consent forms to take part in the study which was conducted online, and they also provided demographic information.

Working group members were informed that in order to recommend an area of research as a priority, it had to meet the following criteria:

i. Research is urgently needed in that area.
ii. There is a gap in the existing evidence in the area, or there is no evidence in existence.
iii. Research in the area will impact, either directly or indirectly, on the quality of life of children requiring palliative care and their families.

Following content analysis, similar responses from working group members were grouped together to ensure that the questionnaire was not repetitive and could be easily completed. Content analysis was undertaken in order to identify themes for research and reduce the number of items to a manageable number for the Delphi process [27, 28]. Content analysis was undertaken manually by one of the researchers and involved a process of categorising and conceptualising the responses. In order to maintain methodological rigor, and due to the fact that manual content analysis has the potential to introduce researcher bias [28–30], the content analysis was emailed to the working group who commented on the analysis and agreed on the items to go forward to the Delphi process.

- Step 2: List of participants

Once the initial list of priorities was created, invitations to participate in a survey were sent to all ICPCN members and ICPCN board members, who were also asked to recommend individuals in their countries/regions that could complete the survey. This sampling technique was used with the goal of having a geographically balanced group of respondents, including participants from developed and developing countries.

- Step 3: Delphi process

A list of 70 research priorities suggested in the first step were put through two rounds of the Delphi process. An online survey was set up using SurveyMonkey, through which participants could access information anonymously and rate the suggested research priorities.

Participants were asked to place an X in the box which they felt best described how important the research topic is. The response choices were:

1. Top priority – urgently needs to be done within the next 24 months.
2. High Priority – needs to be done within the next 24–48 months;
3. Medium priority – needs to be done within the next 4–6 years;
4. Low priority – needs to be done but can wait.
5. Not a priority

Respondents were also given a ‘*Do not know/Not sure*’ option if they did not feel comfortable rating the priority. Information as to how the list of suggested areas for research was built was provided to the participants and an opportunity was given to participants to suggest other areas for research that they strongly felt should be incorporated into the list (Q71. Are there any other areas of global research in CPC that you think should have been included in the list?). Participation in the study was voluntary and completion of the survey was taken as consent to participate. Reminders were sent to participants at fortnightly intervals, along with a reminder through the ICPCN newsletter/website. Summary statistics were run on the results to determine the number of statements that had reached consensus at this stage. Consensus was deemed to have been reached if the percentage of answers scoring 1 (top priority) or 2 (high priority) was above 75 %. It was also agreed that the mean score should be <2 for the area of research to be considered a priority.

The research team reviewed the responses and any additional topics made by the participants in the first round and the initial list of research topics was revised. Those statements that reached consensus during the first round (i.e. >75 % of the participants agreed on the importance) were ‘banked’ prior to round two, as having already gained consensus [30].

The first round of the Delphi process took place between October and November 2012. In the first round of the Delphi, there were a total of 75 questions, divided into 11 sections. Sections 1–10 were based on the broad areas for research and Section 11 was demographic data. There were 70 closed questions i.e. research topics to be prioritised, 1 open question and 4 demographic questions.

In the second round of the Delphi process the responses from the first Delphi round were listed and participants were informed that topics had been removed if 75 % or more of respondents had identified them as a Top or High priority. Several new research topics were included that were identified by participants as missing. The results of the first round of the Delphi process in terms of median, mean and standard deviation were included as information for participants. The same response categories were offered.

The second round of the Delphi took place between December 2012 and January 2013 and had a total of 68 questions, divided into 12 sections. Sections 1–10 were based on the broad areas for research, Section 11 was demographic data and Section 12 asked if they had completed round 1. There were 62 closed questions, 4 demographic questions, a question asking whether they had completed round 1, and a question asking if they would like to receive the results of the study.

- Step 4: Analysis of the ratings

Those areas of research identified as priorities by 75 % or more of the respondents with a mean of <2 after the two rounds of the Delphi process constituted the top priorities for research into CPC. This list was further narrowed down to the top ten according to level of consensus.

## Results

Results of the study have been reported on according to the CHERRIES statement for web-based surveys [31].

### Participants

Twenty invitations to participate in step 1 were sent out and 16 responded (80 %). Of these 16, 3 (19 %) were from low-income countries, 2 (13 %) from lower-middle income countries, 4 (25 %) from upper-middle income countries and 7 (43 %) from high-income countries. Respondents represented a range of professions (doctor, clinical officer, nurse, priest, social worker, and teacher), had been working in CPC for varying lengths of time and worked in eight countries.

The first round of the Delphi process included an ‘open survey’ meaning it was open for each visitor to the survey site and could be forwarded on to others at the discretion of the respondent. Invitations for both rounds of the Delphi study were sent to a total of 758 people. If they chose to participate they were given a unique Identification (ID) number which matched their Internet Protocol (IP) address recorded.

The completion rate, or the number of people agreeing to participate (or submitting the first survey page), divided by the number of people submitting the last questionnaire page [31] was calculated for each round of the Delphi process and can be found in Table 1. A demographic summary of participants can be found in Table 2. 83 of the participants who completed the first round also completed the second round, however 12 (12.6 %) of the participants who completed the second round, had not completed the first Delphi round. Participants resided in 52 countries.

**Table 1:** Completion rate for the Delphi process

1.	Delphi round 1	Number of people submitting the first page	170
		Number of people submitting the last page	153
		Completion rate	90.0 %
2.	Delphi round 2	Number of people submitting the first page	106
		Number of people submitting the last page	95
		Completion rate	89.6 %

**Table 2:** Demographic data for participants

			Round 1 (n = 153)		Round 2 (n = 95)	
1.	Profession	Doctor	70	45.8 %	46	48.4 %
		Clinical Officer	3	2.0 %	4	4.2 %
		Nurse	38	24.8 %	25	26.3 %
		Priest	3	2.0 %	1	1.1 %
		Social Worker	11	7.2 %	5	5.3 %
		Teacher	3	2.0 %	1	1.1 %
		Other	25	16.3 %	13	13.7 %
2.	Region of work	Asia	15	9.8 %	10	10.5 %
		Europe	49	32.0 %	36	37.9 %
		Latin America	17	11.1 %	8	8.4 %
		Middle East	1	0.7 %	0	0 %
		North America	19	12.4 %	13	13.7 %
		Oceania	9	5.9 %	5	5.3 %
		Sub-Saharan Africa	43	28.1 %	23	24.2 %
3.	Main area of work	Clinical care	91	59.5 %	59	62.1 %
		Education	21	13.7 %	13	13.7 %
		Management	10	6.5 %	9	9.5 %
		Policy	8	5.2 %	6	6.3 %
		Research	19	12.4 %	8	8.4 %
		Did not respond	4	2.6 %	-	-

### Results of the different steps in the process

An initial list of 165 areas for research within CPC was identified. The list was refined to 70 through content analysis prior to the first round of the Delphi process.

Results from the first round of the Delphi process (Step 3) were reviewed and analysed. Descriptive statistics were run on the data in order to determine the number of statements that had reached consensus and to show the distribution of responses. Consensus was deemed to have been reached if the percentage of answers scoring 1 (top priority) or 2 (high priority) was > 75 % with a mean score <2. The consensus ratings for the different areas for research ranged from 43 % through to 84 %. Seventeen questions (24.3 %) had a consensus rating of >75 %, 41 (58.6 %) between 60 and 75 %, and 12 (17.1 %) under 60 %.

The seventeen questions which reached consensus were excluded from the second Delphi round [30]. Thirty-seven participants included additional areas for research which, following content analysis, were reduced to 9 areas and were added to the second Delphi round.

Results from the second round of the Delphi process were reviewed and analysed. 62 areas for research within CPC were graded on a scale of priority. Summary statistics were run on the results to determine the number of statements that reached consensus.

The consensus ratings for the different areas for research ranged from 35.9 % through to 78.9 %. Nine areas for research reached consensus therefore making a total of 26 areas in total from both Delphi rounds (Table 3).

**Table 3:** Areas of research that reached consensus as a priority and had a mean < 2

Question	Round in which reached consensus	Percent	Median	Mean	SD
Section 1. Interventions and models of care for CPC					
Interventions and models of care for CPC	1	80.5 %	1	1.76	1.08
Measuring outcomes of care	1	84.1 %	2	1.76	0.93
The challenges to CPC provision	2	77.3 %	2	1.95	1.17
Section 2. Clinical Care					
Assessment of the WHO two-step analgesic ladder for pain management in children (please refer to the new pain guidelines	1	79.4 %	1	1.72	0.96
Use of adjuvant medicines to relive pain	1	75.6 %	2	1.91	0.97
Use of opioids in children	1	77.5 %	1	1.79	1.11
Managing pain in children where there is no morphine (Strong opioids)	1	83.1 %	1	1.61	1.11
Pain management for non-cancer children with chronic life-threatening illness	1	80.0 %	1	1.72	0.99
Understanding the needs of children and their families	1	79.4 %	2	1.83	1
Assessment and management of different symptoms	1	75.0 %	2	1.86	1.09
Non-pharmacological management of pain and other distressing symptoms	2	78.9 %	2	1.91	0.99
Validation of pain assessment tools in different settings/ ages	2	77.8 %	2	1.92	1.04
Perinatal palliative care	2	78.9 %	2	1.91	1.1
Section 3. Psychological issues					
Models of providing psychological care in CPC	1	75.0 %	2	1.92	0.77
Communicating with children and their families	1	80.2 %	2	1.83	0.98
Children's understanding of death and dying	2	78.6 %	2	1.54	1.1
The illness experience for children	2	75.7 %	2	1.96	0.8
Section 7. Policies and Procedures					
Funding for and the cost of CPC	1	84.6 %	1	1.67	0.89
Section 8. Legislation and ethics					
Ethical issues in CPC	1	75.6 %	1	1.8	1.03
Children's rights and palliative care	2	78.2 %	1	1.81	1.13
Section 9. Education					
Training needs for CPC	1	82.7 %	1	1.68	0.96
The impact of education programmes on the provision of CPC	1	75.0 %	2	1.92	0.96
Integration of CPC into core health curriculum	1	79.5 %	1	1.78	0.96
Models of education and training for CPC	2	76.2 %	2	1.89	1.11
Section 10. Other					
The global need for CPC	1	78.6 %	1	1.79	1.15
Assessment of government support for CPC	2	78.2 %	2	1.83	0.94

SD = Standard Deviation

As consensus had been reached on the identification of several priorities, no additional rounds were conducted. The level of agreement on whether an area of research is a priority or not was shown by the percentage of participants who said that the statement was either Top or High priority and the importance of the area of research calculated by the mean, with the lowest mean showing the highest importance. Therefore the priorities for research in CPC as identified through this Delphi study can be seen in Table 4.

**Table 4:** Identified priorities for global research in children’s palliative care

Order of priority	Area of research	Mean (level of importance)	% (level of consensus)
1	Children's understanding of death and dying	1.54	78.6 %
2	Managing pain in children where there is no morphine (Strong opioids)	1.61	83.1 %
3	Funding for and the cost of CPC	1.67	84.6 %
4	Training needs for CPC	1.68	82.7 %
5	Assessment of the WHO two-step analgesic ladder for pain management in children	1.72	79.4 %
5	Pain management for non-cancer children with chronic life-threatening illness	1.72	80.0 %
6	Interventions and models of care for CPC	1.76	80.5 %
6	Measuring outcomes of care	1.76	84.1 %
7	Integration of CPC into core health curriculum	1.78	79.5 %
8	Use of opioids in children	1.79	77.5 %
8	The global need for CPC	1.79	78.6 %
9	Ethical issues in CPC	1.80	75.6 %
10	Children's rights and palliative care	1.81	78.2 %
11	Understanding the needs of children and their families	1.83	79.4 %
11	Communicating with children and their families	1.83	80.2 %
11	Assessment of government support for CPC	1.83	78.2 %
12	Assessment and management of different symptoms	1.86	75.0 %
13	Models of education and training for CPC	1.89	76.2 %
14	Use of adjuvant medicines to relive pain	1.91	75.6 %
14	Non-pharmacological management of pain and other distressing symptoms	1.91	78.9 %
14	Perinatal palliative care	1.91	78.9 %
15	Validation of pain assessment tools in different settings/ ages	1.92	77.8 %
15	Models of providing psychological care in CPC	1.92	75.0 %
15	The impact of education programmes on the provision of CPC	1.92	75.0 %
16	The challenges to CPC provision	1.95	77.3 %
17	The illness experience for children	1.96	75.7 %

It is important to note that whilst the majority of the top 26 priorities for research into CPC are clinical (10/25), none of them fit into the categories of social, spiritual or management and human resource issues, whereas 4, (15.4 %) came under education, 3 (11.5 %) interventions and models of care, 2 (7.7 %) for both legislation and ethics and other, and 1 (3.8 %) under policies and procedures.

When narrowing it down to the top 10 research priorities for children identified through this Delphi study, it can be seen that they are linked to psychological issues, clinical care, policies and procedures, interventions and models of care, education and legislation and ethics (Table 5).

**Table 5:** Top ten priorities by category

Research priorities		Broad research category
1	Children's understanding of death and dying	Psychological issues
2	Managing pain in children where there is no morphine (Strong opioids)	Clinical care
3	Funding for and the cost of CPC	Policies and Procedures
4	Training needs for CPC	Education
5	Assessment of the WHO two-step analgesic ladder for pain management in children	Clinical care
5	Pain management for non-cancer children with chronic life-threatening illness	Clinical care
6	Interventions and models of care for CPC	Interventions and models of care
6	Measuring outcomes of care	Interventions and models of care
7	Integration of CPC into core health curriculum	Education
8	Use of opioids in children	Clinical care
8	The global need for CPC	Other
9	Ethical issues in CPC	Legislation and ethics
10	Children's rights and palliative care	Legislation and ethics

## Discussion and conclusion

The aim of this research was to identify and prioritise research areas in CPC. Although this exercise has been done in the past, it has been focused at the national level and not been able to comment on global alignment or variation in the field. Contributions of this study to the literature are described below.

Participants in this Delphi study represented more than 50 countries and all regions of the world. From the results it is clear that there is consensus on what is important in CPC and that these priorities were chosen independently by the participants. This is important because oftentimes consensus is reached through round table discussions and there might be a tendency for those from the developing countries to have more input since they would by default have more experience in conducting research. By using this method, with this sample, we are able to identify a list of priorities where each respondent received equal weighting of input.

Focusing on the results from the top 10 priorities is interesting. When broken down into broad research categories, there is no singular category that overwhelmed the other, although clinical care had the most priorities. It is not surprising that all four clinical issues were around pain management. It is well documented that treating children’s pain is difficult since not many providers receive formal training, there are myths surrounding the use of opioids, availability of pain medication can be a problem, and many of the pain treatment regimens are not evidence based and/or were developed for adults [32]. This is not to say that there has been no progress in pain management for children. Pain scales have been developed and validated, non-pharmacological methods for treating pain are becoming more widespread, guidelines have been developed [33] and evidence on how pain medications are metabolized in a child’s body are just some of the important evidence. Research in this area is also expensive as it often is driven by large scale clinical trials and the WHO has called for more research into pain management in children [33, 34]. Oftentimes developed countries subsidise the world’s knowledge as they conduct a large proportion of research. Going forward organisations will need to continue to come back to the collaborative model which will provide more equitable answers to pressing questions about pain.

Children’s understanding of death and dying was the top priority and lends itself well to multidisciplinary research. A child’s understanding will depend on a variety of factors including their age and developmental stage, their illness, how long they have been unwell, their experience of death in the family, and their culture [35]. Much of the literature on children’s understanding of death and dying has been based on the developmental perspective, with a child’s understanding of death moving along a linear process. However, it is thought to be more complex than this, with some children having more sophisticated views of death and dying than others, dependent on other issues including social, cultural, personal, and emotional issues [35], alongside their experience of illness [36]. Several studies have addressed issues of religious and cultural background, supporting the view that children’s understanding of death and dying is impacted by religious background, e.g. Christianity, Buddhism, Shintoism [37] and Islam [38, 39]. Likewise differences were seen between Chinese compared to American children [40]. Thus, factors other than age, play an important part in children’s understanding of death and dying and understanding these issues is confronted in sociology, religion, communication, philosophy, child development, and health care. Moving from single discipline and culture studies, to multiple, along with updating the evidence base, is important to understanding how children develop these ideas, communicate them, and act out their feelings. Health care may also benefit from more evidence as they play a vital role in caring for the child and the family. Knowing when to intervene when a child is struggling with this understanding and whom to ask for help is not always obvious.

For the two educational priorities, some progress has been made. While there are many accredited palliative care training programmes, many of them are costly. ICPCN recently developed seven online training modules in CPC. Participants can learn about CPC including issues on pain and symptom management, communicating with children and emotional issues, child development and play, end-of-life care, and grief and bereavement, and receive a certificate once they have completed the courses. Admittedly, these modules are limited in scope and language, although they are currently available in English, Spanish, French, Portuguese, Serbian, Russian, Dutch and Mandarin. However, results from this survey can be used to develop new modules in the areas identified as priorities for research such as models of care. Other organisations can also benefit from the result of this study, which can inform their education agenda. Revising the curriculum is a long-term goal that will require coordination between educational institutions, accrediting agencies, and the health care labour market.

When comparing the results of this international study with those done in Scotland [16, 17] Canada [18] and the USA, some similarities exist. For example pain and symptom control, education and the palliative care needs of children are overlapping themes, yet whilst this might be the case in terms of overall themes, particular areas for research include some of those more traditionally seen as issues in low-resource settings e.g. managing pain in children where there is no morphine, the use of opioids and funding for and the cost of CPC. Other core components identified in the international research and not seen so much in the national research, included interventions and models of care for CPC, the global need for CPC, and, ethical issues in CPC. All of these link into the international, rather than national agenda, and such differences were to be expected.

As with any Delphi study, there are limitations. During the initial stage of identifying research priorities, content analysis was used and this can introduce bias as the researcher does not have the opportunity to discuss their responses directly with participants. To mitigate this the content analysis was shared with working group members who commented on it and changes were made as appropriate. The stability of responses within the study is an important factor, and in particular what happens between Delphi rounds, however as there are no direct ethical issues in this study with regards to the identified priorities, this is not considered to be a significant issue. Parents and patients were not invited to participate although their opinions are certainly important in setting priorities and it is hoped that the opportunity will arise to look at these priorities with an international parent organisation. Finally, no definitions were provided and respondents were left to interpret terms on their own.

Despite these limitations, this is the first study to prioritise research needs in CPC. National and international organisations can look to this list for guidance and confirmation as they work through their own initiatives. Listing the priorities is only the first step in a long agenda that must be carefully planned and executed and more work needs to be done in order to ascertain the differences in research priorities between high and low resource settings, or between different continents. As we move forward in making strides in CPC research, it is crucial that we stay family-focused and drive our decisions by evidence when possible.

## Abbreviations

CHERRIES: The Checklist for Reporting Results of Internet E-Surveys
CPC: Children’s Palliative Care
ICPCN: International Children’s Palliative Care Network
ID: Identification
IP: Internet Protocol
SSA: Sub-Saharan Africa
UICC: Union Internationale Contre le Cancer
WHO: World Health Organization
